# Supplementary figures and images for: Genetic ablation of interleukin-17A augments fibrosis in a mouse model of cholestatic liver injury
Source: PLoS One. 2026 Feb 6;21(2):e0342251. doi: 10.1371/journal.pone.0342251 (PMC12880643; doi:10.1371/journal.pone.0342251)

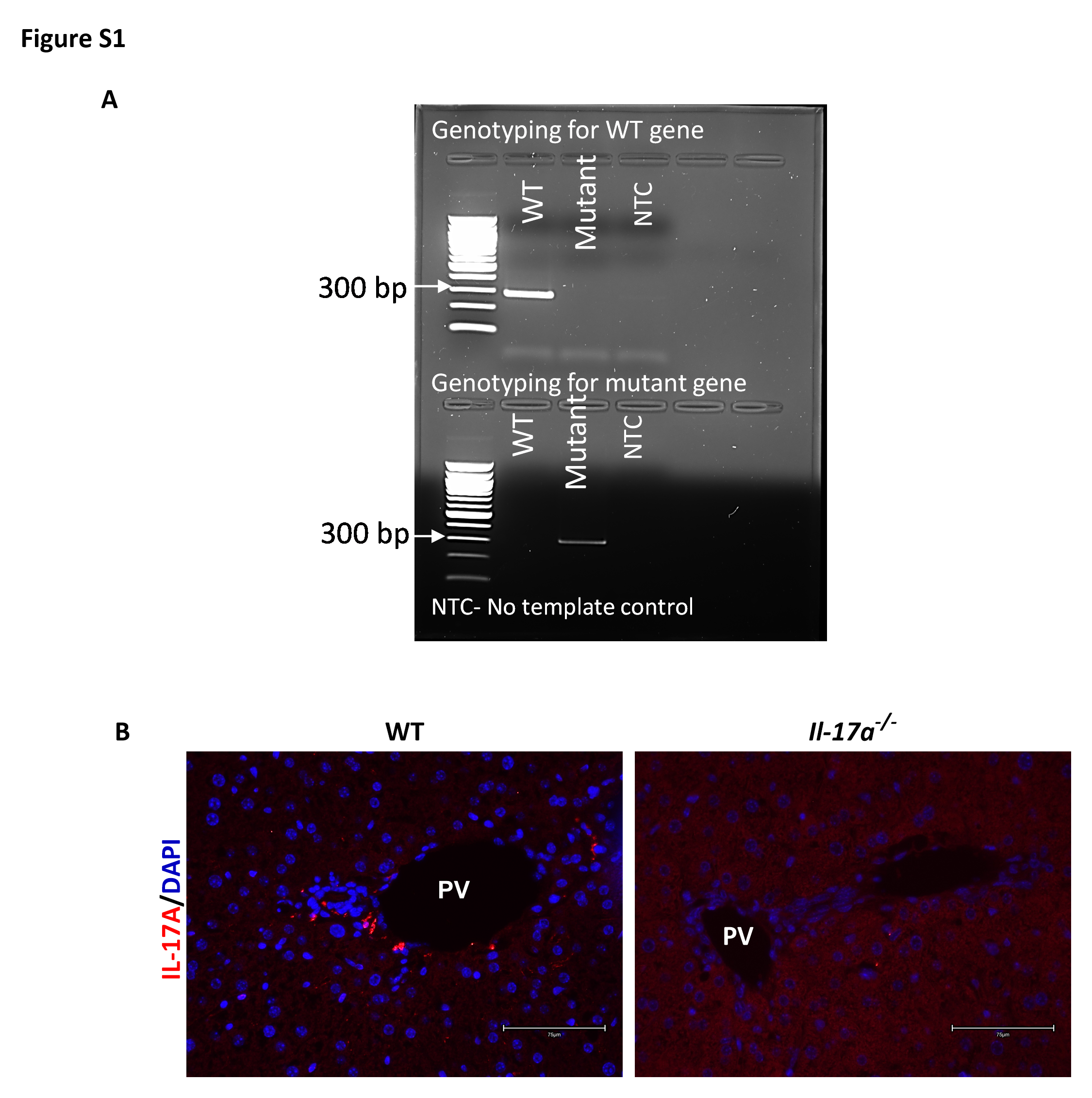

Supplement: S1 Fig — (TIF) [file pone.0342251.s002.tif]

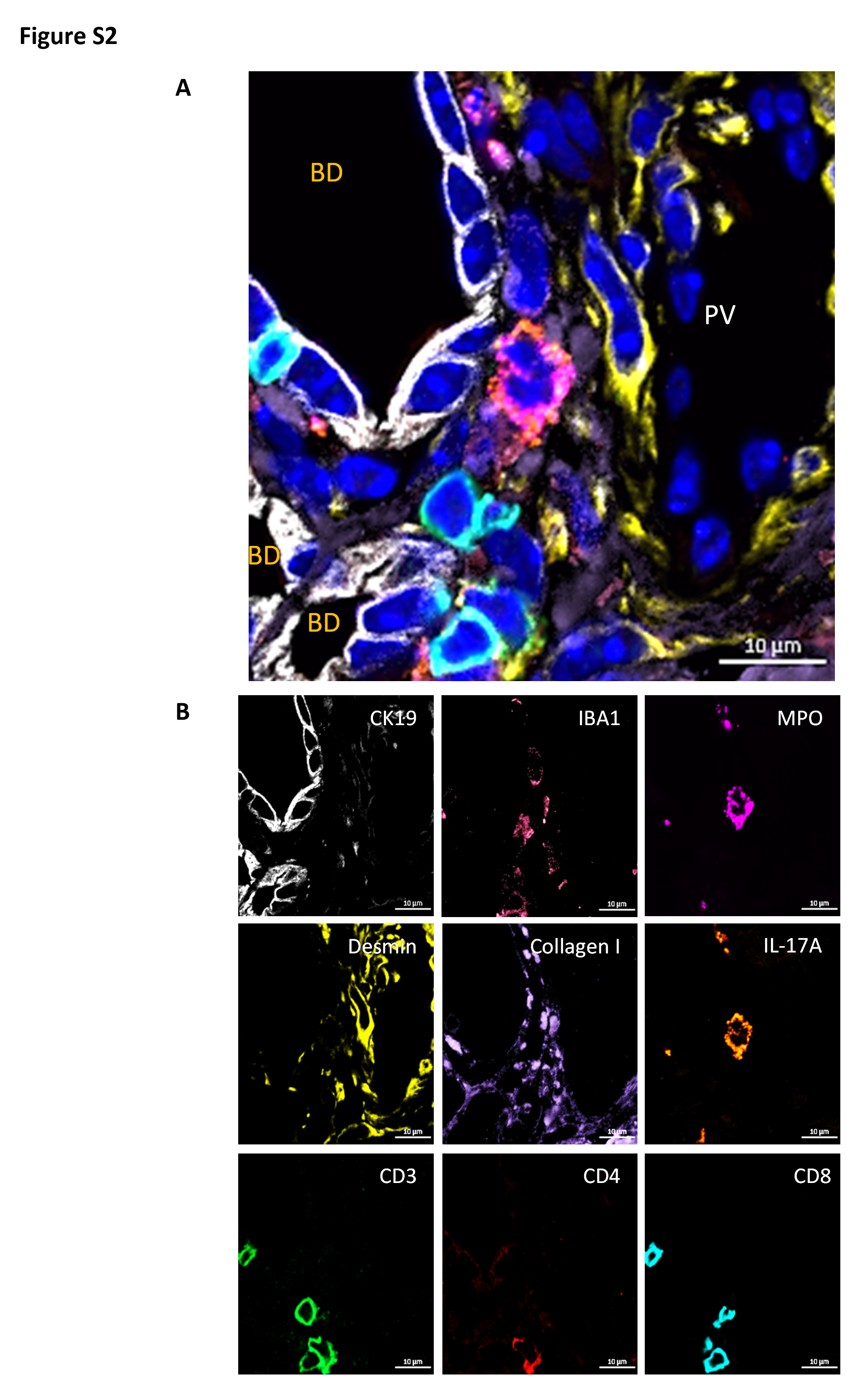

Supplement: S2 Fig — (TIF) [file pone.0342251.s003.tif]

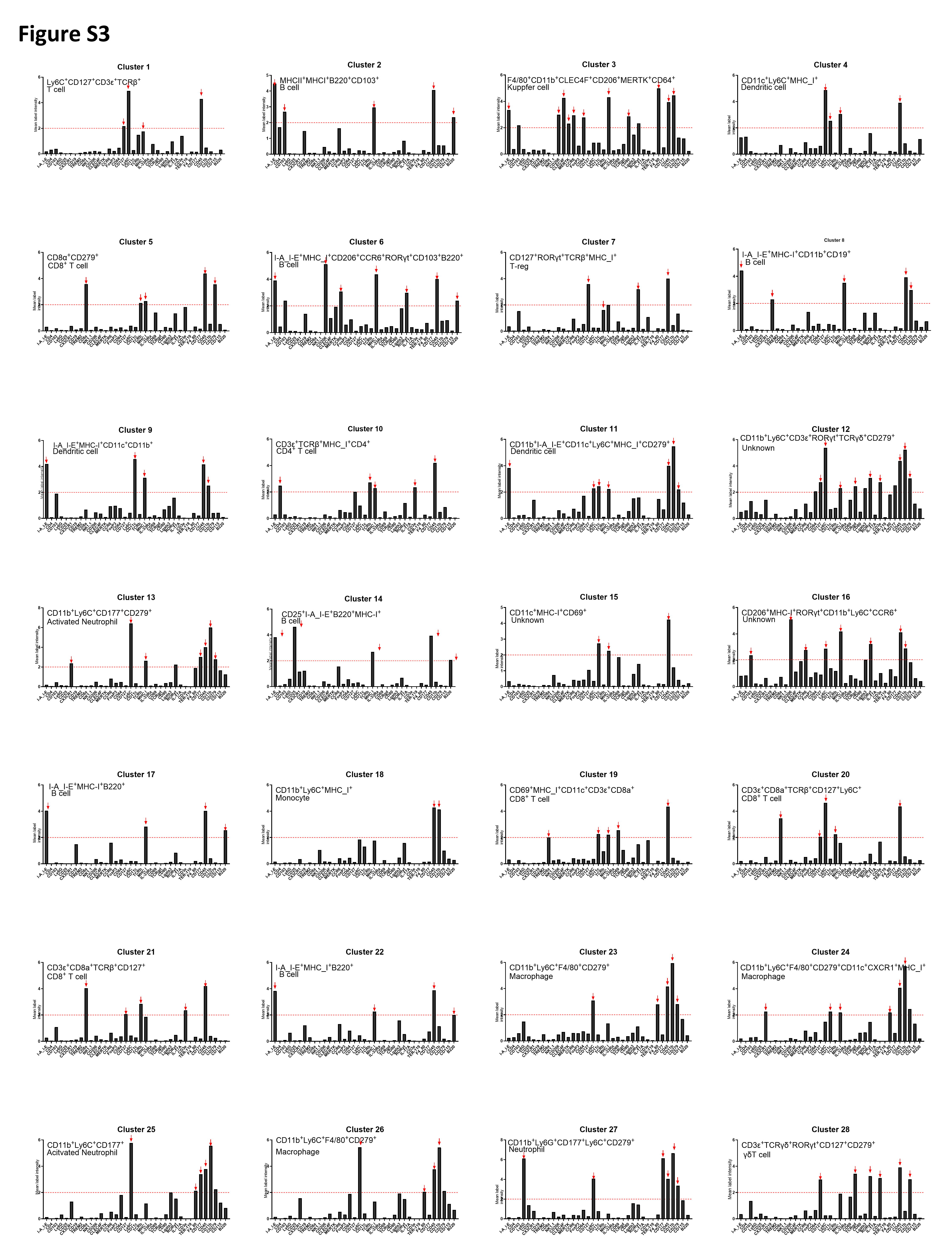

Supplement: S3 Fig — (TIF) [file pone.0342251.s004.tif]

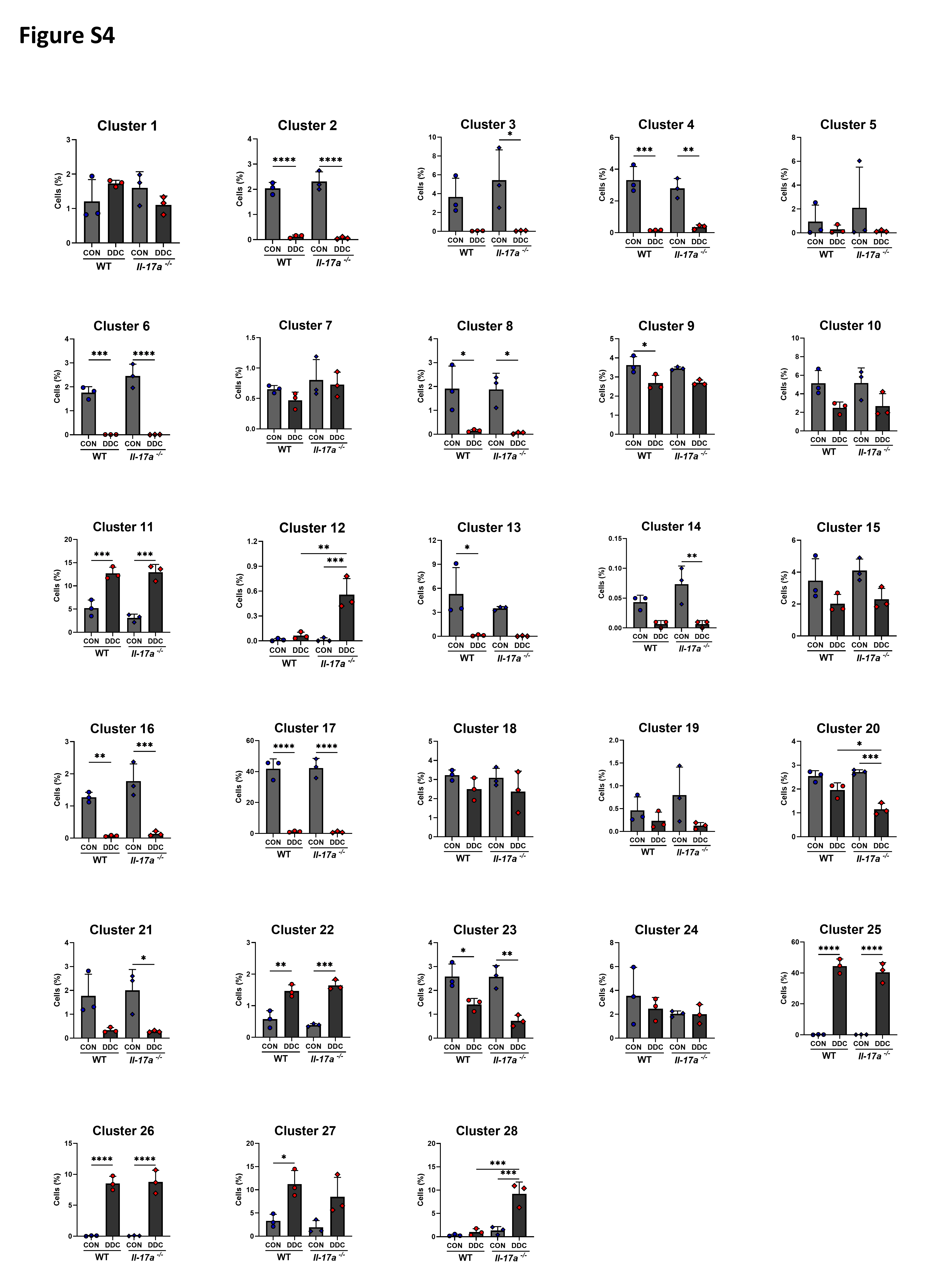

Supplement: S4 Fig — (TIF) [file pone.0342251.s005.tif]

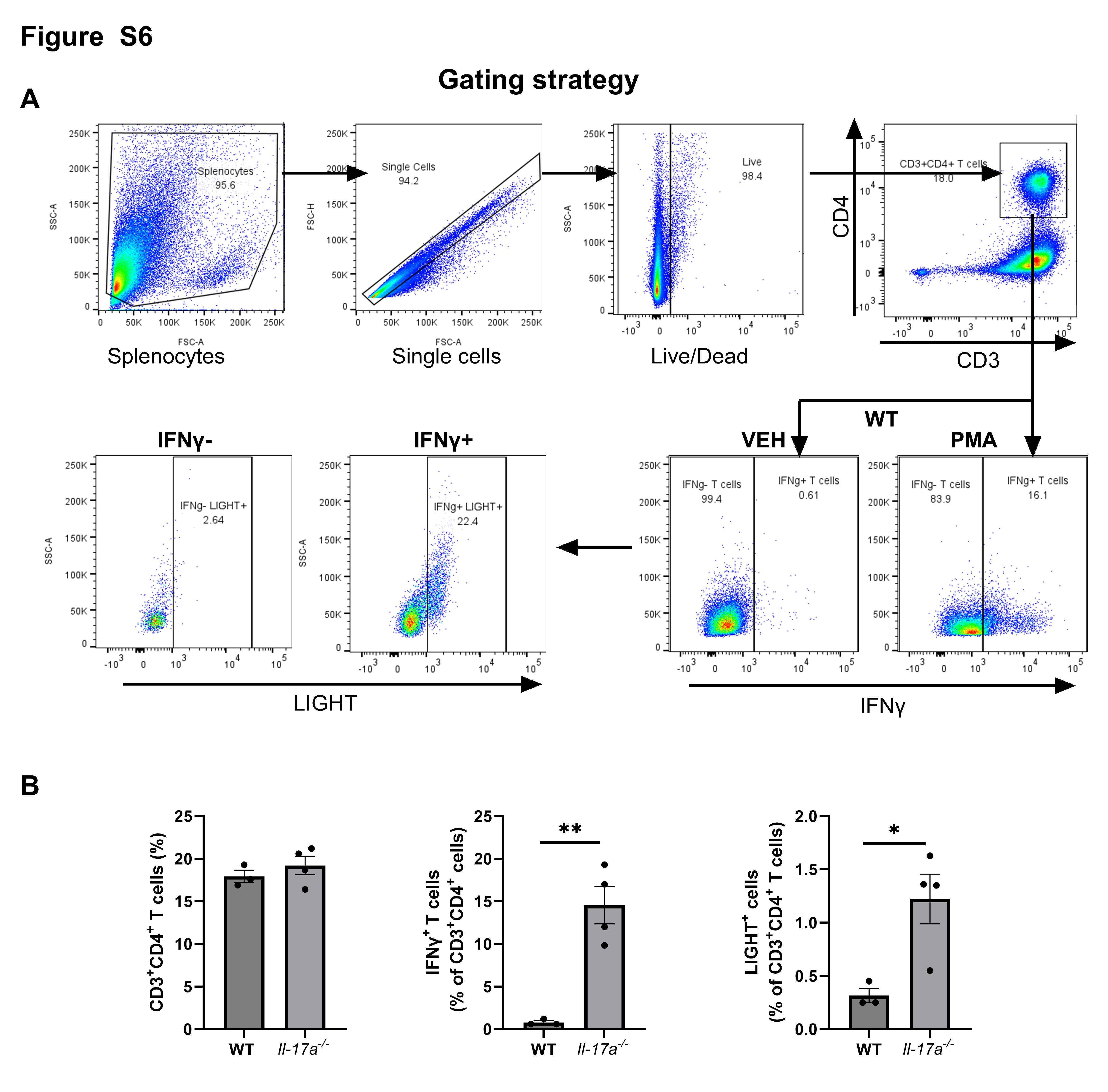

Supplement: S6 Fig — (TIF) [file pone.0342251.s007.tif]
