# Supplementary material for: Genetic ablation of interleukin-17A augments fibrosis in a mouse model of cholestatic liver injury
Source: PLoS One. 2026 Feb 6;21(2):e0342251. doi: 10.1371/journal.pone.0342251 (PMC12880643; doi:10.1371/journal.pone.0342251)
Supplement: S1 Table — (DOCX) [file pone.0342251.s008.docx]

**Table S1. List of antibodies used for immunofluorescent staining of tissue sections and for flow cytometry**

| **Antibody** | **Catalog #** | **Manufacturer** | **Clone** | **Host species** | **Dilution** |
| --- | --- | --- | --- | --- | --- |
| **Primary antibodies** | | | | | |
| CD3 | 14-0032-82 | ThermoFisher Scientific | 17A2 | Rat | 1:100 |
| CD4 | 14-9766-82 | ThermoFisher Scientific | 4SM95 | Rat | 1:100 |
| CD8 | 98941 | Cell signaling | D4W2Z | Rabbit | 1:100 |
| CK19 | 12434S | Cell signaling | D4G2 | Rabbit | 1:100 |
| CK7 | Sc-53623 | Santa Cruz | LP1K | Mouse | 1:100 |
| Collagen I | 1310-01 | Southern BIotech | Polyclonal | Goat | 1:100 |
| Desmin | RB9014PO | ThermoFisher Scientific | Polyclonal | Rabbit | 1:200 |
| Desmin | 5332 | Cell signaling | D93F5 | Rabbit | 1:100 |
| IBA1 | 100-1028 | Novus Bio | Polyclonal | Goat | 1:200 |
| IL-17A | 13838S | Cell signaling | D1X7L | Rabbit | 1:100 |
| MPO | AF3667 | R&D Systems | Polyclonal | Goat | 1:200 |
| **Secondary antibodies** | | | | | |
| Anti-Rat AF568 | A11077 | ThermoFisher Scientific | - | Goat | 1:200 |
| Anti-Rabbit AF594 | A32754 | ThermoFisher Scientific | - | Donkey | 1:200 |
| Anti-Rabbit AF488 | A21206 | ThermoFisher Scientific | - | Donkey | 1:200 |
| Anti-Goat AF568 | A11057 | ThermoFisher Scientific | - | Donkey | 1:200 |
| Anti-Goat AF488 | A21467 | ThermoFisher Scientific | - | Chicken | 1:200 |
| Anti-Mouse AG568 | A11004 | ThermoFisher Scientific | - | Goat | 1:200 |
|  | | | | | |
| **Antibodies for Flow cytometry** | | | | | |
| CD45 FITC | 130-110-796 | Miltenyi | REA737 | - | 1:50 |
| CD3 FITC | 130-119-798 | Miltenyi | REA641 | - | 1:50 |
| CD3 PE | 130-121-133 | Miltenyi | REA641 | - | 1:50 |
| CD4 Viogreen | 130-123-899 | Miltenyi | GK1.5 | - | 1:50 |
| CD4 BV510 | 100449 | Biolegend | GK1.5 | - | 1:50 |
| CD8a PE | 130-102-807 | Miltenyi | 53-6.7 | - | 1:10 |
| NK1.1 PE | 130-116-504 | Miltenyi | PK136 | - | 1:50 |
| CD11b PE | 130-113-806 | Miltenyi | REA592 | - | 1:50 |
| Ly-6G PE | 130-119-914 | Miltenyi | REAL176 | - | 1:50 |
| CD11c PE | 130-110-838 | Miltenyi | REA754 | - | 1:50 |
| LIGHT/TNFSF14 AF405 | FAB17942V | R&D System | 885310 | - | 1:50 |
| FOXP3 PE | 320008 | Biolegend | 150D | - | 1:50 |
| T-bet PE | 644809 | Biolegend | 4B10 | - | 1:50 |
| GATA3 PE | 12-9966-42 | Invitrogen | TWAJ | - | 1:50 |
| RORγt PE | 12-6981-82 | Invitrogen | B2D | - | 1:50 |
| IFNγ PE-Cy7 | 505825 | Biolegend | XMg1.2 | - | 1:50 |
| CD4 PE | 100408 | Biolegend | GK1.5 | - | 1:50 |
| CD3 FITC | 100204 | Biolegend | 17A2 | - | 1:50 |
