# Supplementary material for: Genetic ablation of interleukin-17A augments fibrosis in a mouse model of cholestatic liver injury
Source: PLoS One. 2026 Feb 6;21(2):e0342251. doi: 10.1371/journal.pone.0342251 (PMC12880643; doi:10.1371/journal.pone.0342251)
Supplement: S2 Table — (DOCX) [file pone.0342251.s009.docx]

**Table S2. List of primers used for quantitative real-time PCR.**

| Gene ID | Forward primer sequence | Reverse primer sequence |
| --- | --- | --- |
| **Mouse** |  |  |
| *Tgfβ1* | TGATACGCCTGAGTGGCTGTCT | CACAAGAGCAGTGAGCGCTGAA |
| *Timp1* | TCTTGGTTCCCTGGCGTACTCT | GTGAGTGTCACTCTCCAGTTTGC |
| *Fn1* | CCCTATCTCTGATACCGTTGTCC | TGCCGCAACTACTGTGATTCGG |
| *Acta2* | TGCTGACAGAGGCACCACTGAA | CAGTTGTACGTCCAGAGGCATAG |
| *Col1a1* | CCTCAGGGTATTGCTGGACAAC | CAGAAGGACCTTGTTTGCCAGG |
| *Col4a1* | ATGGCTTGCCTGGAGAGATAGG | TGGTTGCCCTTTGAGTCCTGGA |
| *Actin* | CATTGCTGACAGGATGCAGAAGG | TGCTGGAAGGTGGACAGTGAGG |
| *Gapdh* | AGGTCGGTGTGAACGGATTTG | TGTAGACCATGTAGTTGAGGTCA |
| *IL17a* | AAGGCAGCAGCGATCATC | GGAACGGTTGAGGTAGTCTGA |
| *Il17f* | CTGAGGCCCGCAGAC | GCTGAATGGCGACGGAGT |
| *18S* | TCAACACGGGAAACCTCA | CGCTCCACCAACTAAGAACG |
| *Tnfsf14* | CCAGGCTACTTCTGTGAGAACC | CAGTCAGCACATACAGTGTCCTG |
| **Human** |  |  |
| *ACTA2* | GGAGATCACGGCCCTAGCAC | AGGCCCGGCTTCATCGTAT |
| *COL1A1* | GATTCCCTGGACCTAAAGGTGC | AGCCTCTCCATCTTTGCCAGC |
| *COL4A1* | GGA CTA CCT GGA ACA AAA GGG | GCC AAG TAT CTC ACC TGG ATC A |
| *PDGFRA* | TGGCAGTACCCCATGTCTGAA | CCAAGACCGTCACAAAAAGGC |
