# Supplementary material for: Genetic ablation of interleukin-17A augments fibrosis in a mouse model of cholestatic liver injury
Source: PLoS One. 2026 Feb 6;21(2):e0342251. doi: 10.1371/journal.pone.0342251 (PMC12880643; doi:10.1371/journal.pone.0342251)
Supplement: S3 Table — (DOCX) [file pone.0342251.s010.docx]

**Table S3. List of antibodies and clones used for mass cytometry**

|  | **Antigen** | **Clone** | **Company** | **Catalog Number** | **Label** |
| --- | --- | --- | --- | --- | --- |
| 1 | CD45 | 30-F11 | FDM | 3089005B | 089Y |
| 2 | CD4 | RM4-5 | Biolegend | 100561 | 112Cd |
| 3 | CD103 | 20000000 | Biolegend | 121402 | 114Cd |
| 4 | Galectin-3 | 202213 | R&D | MAB1197 | 141Pr |
| 5 | CD11c | N418 | FDM | 3142003B | 142Nd |
| 6 | TCRb | H57-597 | FDM | 3143010B | 143Nd |
| 7 | MHC I | 28-14-8 | FDM | 3144016B | 144Nd |
| 8 | IL-33Ra | D1J9 | Biolegend | 145302 | 145Nd |
| 9 | CD69 | H1.2F3 | FDM | 3145005B | 146Nd |
| 10 | RORγt | B2D | Abcam | ab232516 | 147Sm |
| 11 | TCR gd | GL3 | Biolegend | 118101 | 148Nd |
| 12 | IL-17A | TC11-18H10.1 | FCM | 3174002B | 149Sm |
| 13 | MHC II | M5/114.15.2 | Biolegend | 107637 | 150Nd |
| 14 | CD206 | C068C2 | Biolegend | 141702 | 151Eu |
| 15 | CD3e | 145-2C11 | FDM | 3152004B | 152Sm |
| 16 | CLEC4F |  | R&D | AF2784 | 153Eu |
| 17 | TER-119 | TER-119 | FDM | 3154005B | 154Sm |
| 18 | MERTK | 108928 | R&D | MAB5912 | 155Gd |
| 19 | CCR6 | 29-2L17 | FDM | 3156016A | 156Gd |
| 20 | FoxP3 | FJK-16s | FCM | 3158003A | 158Gd |
| 21 | F4/80 | BM8 | FDM | 3159009B | 159Tb |
| 22 | CD64 | 290322 | R&D | MAB20741 | 160Gd |
| 23 | LY6G | 1A8 | Biolegend | 127637 | 161Dy |
| 24 | CD25 | 3C7 | FDM | 3151002B | 163Dy |
| 25 | CX3CR1 | SA011F11 | FDM | 3164023B | 164Dy |
| 26 | CD127 | A7R34 | Biolegend | 135029 | 165Ho |
| 27 | CD19 | 6D5 | FDM | 3166015B | 166Er |
| 28 | TREM2 | 237920 | R&D | MAB17291 | 167Er |
| 29 | CD8a | 53-6.7 | FDM | 3168003B | 168Er |
| 30 | CD177 | 1171A | R&D | MAB8186 | 169Tm |
| 31 | NK1.1 | PK136 | FDM | 3170002B | 170Er |
| 32 | CD80 | 16-10A1 | FDM | 3171008B | 171Yb |
| 33 | CD11b | M1/170 | FDM | 3172012B | 172Yb |
| 34 | CD279 | RMP1-30 | FDM | 3159024B | 173Yb |
| 35 | CD115 | AFS98 | Biolegend | 135521 | 174Yb |
| 36 | LY6C | HK1.4 | Biolegend | 128039 | 175Lu |
| 37 | CD45R | RA3-6B2 | FDM | 3176002B | 176Yb |
