# Supplementary material for: Genetic ablation of interleukin-17A augments fibrosis in a mouse model of cholestatic liver injury
Source: PLoS One. 2026 Feb 6;21(2):e0342251. doi: 10.1371/journal.pone.0342251 (PMC12880643; doi:10.1371/journal.pone.0342251)
Supplement: Attachment — Submitted filename: Response to reviewers.docx [file pone.0342251.s011.docx]

**POINTS TO ADDRESS:**

When submitting your revision, we need you to address these additional requirements.

1.Please ensure that your manuscript meets PLOS ONE's style requirements, including those for file naming. The PLOS ONE style templates can be found at

[https://journals.plos.org/plosone/s/file?id=wjVg/PLOSOne_formatting_sample_main_body.pdf](https://nam12.safelinks.protection.outlook.com/?url=https%3A%2F%2Fjournals.plos.org%2Fplosone%2Fs%2Ffile%3Fid%3DwjVg%2FPLOSOne_formatting_sample_main_body.pdf&data=05%7C02%7CHoover.Courtney%40mayo.edu%7C2beb4a547b2b473b667208de0fd312d5%7Ca25fff9c3f634fb29a8ad9bdd0321f9a%7C0%7C0%7C638965596376021500%7CUnknown%7CTWFpbGZsb3d8eyJFbXB0eU1hcGkiOnRydWUsIlYiOiIwLjAuMDAwMCIsIlAiOiJXaW4zMiIsIkFOIjoiTWFpbCIsIldUIjoyfQ%3D%3D%7C0%7C%7C%7C&sdata=3WX5LQvoE7F7swTbsVJ2Ors%2BIhwGsn1Z1ZmdQgebcZU%3D&reserved=0) and

[https://journals.plos.org/plosone/s/file?id=ba62/PLOSOne_formatting_sample_title_authors_affiliations.pdf](https://nam12.safelinks.protection.outlook.com/?url=https%3A%2F%2Ftrack.editorialmanager.com%2FCL0%2Fhttps%3A%252F%252Fjournals.plos.org%252Fplosone%252Fs%252Ffile%253Fid%3Dba62%252FPLOSOne_formatting_sample_title_authors_affiliations.pdf%2F1%2F010f019a00ef6917-1d1dc957-689f-4e63-8bca-c168ebb57451-000000%2FxVSy2hH03deIEd925ztLjG1QZDiZlN2rrSHSSgb_7-w%3D233&data=05%7C02%7CHoover.Courtney%40mayo.edu%7C2beb4a547b2b473b667208de0fd312d5%7Ca25fff9c3f634fb29a8ad9bdd0321f9a%7C0%7C0%7C638965596376032120%7CUnknown%7CTWFpbGZsb3d8eyJFbXB0eU1hcGkiOnRydWUsIlYiOiIwLjAuMDAwMCIsIlAiOiJXaW4zMiIsIkFOIjoiTWFpbCIsIldUIjoyfQ%3D%3D%7C0%7C%7C%7C&sdata=Me1y%2F4RSHzuakUKt288A2ygVghe4gCFDXqYhcPXHIh0%3D&reserved=0)

The manuscript is formatted per the PLOS ONE style requirements.

2. Thank you for stating the following financial disclosure:

“This work was supported by NIDDK-funded grant DK124182 (GJG), the NIDDK-funded Optical Microscopy Core of the Mayo Clinic Center for Cell Signaling in Gastroenterology (P30DK084567), and the Mayo Clinic, Rochester.”

Please state what role the funders took in the study.  If the funders had no role, please state: "The funders had no role in study design, data collection and analysis, decision to publish, or preparation of the manuscript."

Done

3. Please expand the acronym “NIDDK” (as indicated in your financial disclosure) so that it states the name of your funders in full.

The financial disclosure statement has been amended to include the expanded acronym NIDDK (National Institute of Diabetes and Digestive and Kidney Diseases).

4. Thank you for stating the following in your Competing Interests section:

“Disclosures: The authors have no financial or personal disclosures relevant to this manuscript.”

Please complete your Competing Interests on the online submission form to state any Competing Interests. If you have no competing interests, please state "The authors have declared that no competing interests exist.", as detailed online in our guide for authors at [http://journals.plos.org/plosone/s/submit-now](https://nam12.safelinks.protection.outlook.com/?url=http%3A%2F%2Fjournals.plos.org%2Fplosone%2Fs%2Fsubmit-now&data=05%7C02%7CHoover.Courtney%40mayo.edu%7C2beb4a547b2b473b667208de0fd312d5%7Ca25fff9c3f634fb29a8ad9bdd0321f9a%7C0%7C0%7C638965596376057798%7CUnknown%7CTWFpbGZsb3d8eyJFbXB0eU1hcGkiOnRydWUsIlYiOiIwLjAuMDAwMCIsIlAiOiJXaW4zMiIsIkFOIjoiTWFpbCIsIldUIjoyfQ%3D%3D%7C0%7C%7C%7C&sdata=cYwwzFEJFbiC%2FkGoCo02IkLd40Zt0psfAMvZn2b22M0%3D&reserved=0)

 Done

5. We note that your Data Availability Statement is currently as follows: Data supporting the findings of this study are available within the article and in the supplementary information.

Please confirm at this time whether or not your submission contains all raw data required to replicate the results of your study. Authors must share the “minimal data set” for their submission. PLOS defines the minimal data set to consist of the data required to replicate all study findings reported in the article, as well as related metadata and methods ([https://journals.plos.org/plosone/s/data-availability#loc-minimal-data-set-definition](https://nam12.safelinks.protection.outlook.com/?url=https%3A%2F%2Fjournals.plos.org%2Fplosone%2Fs%2Fdata-availability%23loc-minimal-data-set-definition&data=05%7C02%7CHoover.Courtney%40mayo.edu%7C2beb4a547b2b473b667208de0fd312d5%7Ca25fff9c3f634fb29a8ad9bdd0321f9a%7C0%7C0%7C638965596376075310%7CUnknown%7CTWFpbGZsb3d8eyJFbXB0eU1hcGkiOnRydWUsIlYiOiIwLjAuMDAwMCIsIlAiOiJXaW4zMiIsIkFOIjoiTWFpbCIsIldUIjoyfQ%3D%3D%7C0%7C%7C%7C&sdata=ItTwJME5noHNITyCWhgsJGBgDz1Y0OCccwqqQRTwURE%3D&reserved=0)).

For example, authors should submit the following data:

- The values behind the means, standard deviations and other measures reported;

- The values used to build graphs;

- The points extracted from images for analysis.

Authors do not need to submit their entire data set if only a portion of the data was used in the reported study.

Please see uploaded excel spread sheet.

If your submission does not contain these data, please either upload them as Supporting Information files or deposit them to a stable, public repository and provide us with the relevant URLs, DOIs, or accession numbers. For a list of recommended repositories, please see [https://journals.plos.org/plosone/s/recommended-repositories](https://nam12.safelinks.protection.outlook.com/?url=https%3A%2F%2Fjournals.plos.org%2Fplosone%2Fs%2Frecommended-repositories&data=05%7C02%7CHoover.Courtney%40mayo.edu%7C2beb4a547b2b473b667208de0fd312d5%7Ca25fff9c3f634fb29a8ad9bdd0321f9a%7C0%7C0%7C638965596376087759%7CUnknown%7CTWFpbGZsb3d8eyJFbXB0eU1hcGkiOnRydWUsIlYiOiIwLjAuMDAwMCIsIlAiOiJXaW4zMiIsIkFOIjoiTWFpbCIsIldUIjoyfQ%3D%3D%7C0%7C%7C%7C&sdata=msKeTTukKYhh%2F8xSX%2BFqWFSsCXLMHSnklDqQIOYTAvA%3D&reserved=0).

If there are ethical or legal restrictions on sharing a de-identified data set, please explain them in detail (e.g., data contain potentially sensitive information, data are owned by a third-party organization, etc.) and who has imposed them (e.g., an ethics committee). Please also provide contact information for a data access committee, ethics committee, or other institutional body to which data requests may be sent. If data are owned by a third party, please indicate how others may request data access.

Not applicable

6. If the reviewer comments include a recommendation to cite specific previously published works, please review and evaluate these publications to determine whether they are relevant and should be cited. There is no requirement to cite these works unless the editor has indicated otherwise. 

Additional Editor Comments:

As you can see, both reviewers appreciated your work, I am looking forward to the revised version!

**RESPONSES TO COMMENTS BY REVIEWER NUMBER 1**

We thank the reviewer for his/her thoughtful and constructive examination of our manuscript. We have addressed all the reviewer’s concerns in detail. Our responses to the reviewer’s comments are as follows:

**COMMENT 1**. Used model possesses critical limitations that undermine translational relevance. The study exclusively uses the DDC model, which does not truly model human cholangiopathies like PSC or PBC. It primarily induces bile duct proliferation and periductal fibrosis, but lacks the chronicity and immune features (e.g., autoantibodies, IBD comorbidity) found in PSC. No validation in a second model (e.g., BDL, Mdr2-/-) is a serious oversight, especially since the authors make clinical inferences about anti-IL-17 therapy in PSC. The discussion acknowledges this limitation, but this should not just be noted — it weakens the core claims of the manuscript.

**RESPONSE.** We appreciate the reviewer’s perspective regarding murine models of cholestatic liver injury and human relevance to the cholangiopathies and agree that there are no perfect models which recapitulate the associated inflammatory bowel disease, autoantibodies, chronicity, propensity to bile duct cancer, cirrhosis, etc. observed in PSC. The bile duct ligation model is only a three-week model and lethal in our experience (PMID: 19051025), whereas the *Mdr2^-/-^* model is a hepatocyte genetic model and humans with the defect do not develop a PSC phenotype. As nicely written by the reviewer, the DDC model does elicit bile duct proliferation and periductal fibrosis prominent features of PSC and other cholestatic liver disease (PMID: 39251168). Given our interest in the ductular reaction and periductal fibrosis, the DDC model is our preferred model for investigating these critical features of cholestatic liver diseases including PSC. We have provided a stronger rationale for utilizing this model in the revised manuscript (lines: 412-416).

**COMMENT** 2. In fact, LIGHT mechanism is not directly demonstrated in vivo. LIGHT expression is shown to be upregulated in CD4+ Th1 cells in flow cytometry, and LIGHT can activate HSCs in vitro — but no causal link is shown in vivo. There is no use of LIGHT-deficient mice, blocking antibodies, or neutralization strategies. No functional experiment demonstrates that the fibrosis seen in Il-17a-/- mice is actually due to LIGHT — this is speculative at best. A correlative upregulation is not a mechanistic demonstration.

**RESPONSE.** We agree with the gist of the reviewer’s comments. However, there are no conditional knockout mice for LIGHT and in the absence of specific cell type deficiency, it would be challenging to address an explicit causal relationship between cell types with reference to LIGHT signaling. In the discussion, we note that LIGHT-induced fibrosis is inferred but not mechanistically elucidated in our studies and a direct conclusive mechanistic link between the observations is lacking and will require development of a conditional knockout mouse (lines: 433-435).

**COMMENT** 3. IL-17A is known to have complex, context-dependent roles in mucosal immunity and fibrosis. The authors cite this but still oversimplify by declaring IL-17A as "restraining" fibrosis via LIGHT regulation. Th1 skewing is assumed based on increased LIGHT in Th1 cells, but no cytokine profile (e.g., IFNγ, IL-2) is provided to support Th1 dominance. Other CD4+ subsets (e.g., Th17, Treg) are shown to express LIGHT, but the authors arbitrarily focus on Th1 cells.

**RESPONSE.** We agree that IL-17A has context-dependent roles and have revised the text to better reflect this complexity, avoiding any implication that IL-17A **uniformly** restrains fibrosis. Our data supports a model in which IL-17A modulates fibrosis in cholestasis at least in part through regulation of LIGHT expression by Th1 CD4+ T cells, and we now state this more cautiously. We provide clarification that we observe LIGHT expression to be enriched (but not exclusively) in Th1 cells. Although other CD4 T cells subsets express LIGHT, our focus on Th1 CD4+ T cells is based on the premise that Th1 biased CD4 T cells are the major effector populations in multiple murine models of liver inflammation (PMID 37832383, 40631180, 39375367, 22906987). T-bet (Tbx21) is widely accepted as the lineage-defining transcription factor and surrogate marker for Th1 cells, and is commonly used to identify Th1 cells in murine tissues when multiplex cytokine staining is not feasible, such as in the DDC model where porphyrin accumulation causes broad spectrum intrinsic autofluorescence with spillover into multiple channels, which precludes the use of large, high-parameter flow cytometry panels.

However, to further confirm enriched LIGHT expression in the Th1 subset we performed flow cytometry on mouse splenocytes and observed that indeed, LIGHT expression is significantly enriched in IFNү^+^ subset of CD4 T cells upon PMA/ ionomycin stimulation (Fig 6G of the revised manuscript and shown below):

Accordingly, the manuscript has been updated in the methods (lines:204-211), and the results sections, (lines: 378-387).

**COMMENT** 4. Although CyTOF is used to generate 28 clusters, the analysis is descriptive and shallow: no statistical clustering (e.g., PCA, UMAP, cluster-specific testing), no deep phenotype mapping beyond broad lineage categories, no correlation between specific clusters and fibrosis severity.

**RESPONSE**. Mass cytometry analysis was performed by the Immune Monitoring core of the institute that also provided bioinformatic support for data analysis. UMAP and t-SNE are both dimension reduction statistical analytical tools used to visualize non-linear datasets to resolve similar gene/protein expressing cells into clusters characterized by unique expression profiles. While UMAP is traditionally used for large transcriptomic datasets, t-SNE mapping is a standard approach for dimension reduction in data sets with limited number of traits and magnitude differences and is thus the preferred choice for immune cell phenotyping. The data that we present in the manuscript is limited to phenotypic characterization that sorted the immune cells into specific clusters but does not provide any transcriptome information that can be parsed for downstream analysis. Furthermore, as mentioned in the manuscript (lines: 302-304), there were no differences in the abundance of any single cluster between the cholestatic WT or mutant mice, thus precluding a correlation between a specific cluster and fibrosis severity. However, total T cells increased in the *Il17a^-/-^* mice with liver injury. These results have been addressed with greater clarity in the manuscript (lines:299-308).

**COMMENT** 5. Many key figures (especially immunostaining) are poorly quantified or not quantified at all. No available description of the sample number per experiment per quantification. No scoring system or blinded analysis is mentioned for fibrosis, desmin, CK19. Quantification of Sirius Red is questionable — was this done per portal area, per field, or whole slide? Quantitative rigor is lacking throughout the manuscript.

**RESPONSE**. The three key histological parameters - ductular reaction, inflammation and fibrosis were analyzed objectively by digital image analysis. For each of these parameters, 2 to 3 markers were used for evaluation. In all cases a minimum of at least ten random images per animal at 10x or 20x magnification were used for analysis, and all quantification was based on area per field of view. At least 10 to 11 mice were used for each analysis. The materials and methods section have been updated to reflect these details (lines: 159-160). These results are presented in Figs 2A, 2B for CK19 and CK7, Figs 3A, B, C for fibrosis, Figs 4E, for macrophages and neutrophils. An excel file including the original data points for generating graphs has been uploaded to the site for reference. Further, as fibrosis was the only parameter that showed the greatest difference between the WT and the mutant mice, we also provide confirmatory data from quantitative real time PCR for several pro-fibrotic genes.

**COMMENT** 6. Key Controls are missing such as: No IL-17F single KO or IL-17A/F double KO controls used in key experiments. No isotype controls shown in flow cytometry for LIGHT or transcription factor staining. No cell-specific knockout models used to prove LIGHT source or action. No cytokine stimulation of CD4+ T cells to show IL-17A suppresses LIGHT.

**RESPONSE**. Depletion of *Il17a* in mice did not result in a compensatory increase in the gene expression of the *Il17f* cytokine. This was determined by gene expression in whole liver, and the results are presented in Fig 1C and clarified in line 233 of the manuscript. DDC diet-induced cholestasis increased fibrosis in the *Il17af^-/-^* as determined by increased gene expression of several pro-fibrotic genes, *Tgfb, Timp1, Fn1, Acta2,* and *Col1a1*. These results are presented in Fig 3D and mentioned on lines 270-271. Collectively, the lack of compensatory *Il-17f* expression*,* and similar fibrosis in the *Il-17a^-/-^* and *Il17af^-/-^* animals suggest that *Il-17f* did not contribute to the DDC diet-induced phenotype.

For the flow cytometry experiment, gates were set using FMO (Fluorescence Minus One) controls. As FMO controls account for the spillover fluorescence from other channels, they provide a more accurate way to set gates than single-stained isotype controls in a multicolor panel (PMID: 32926370). The use of FMO controls for flow cytometry experiments is clarified on lines 201-202.

To address the comment on IL-17A restraining LIGHT, we compared the CD3^+^CD4^+^ T cell populations in WT and *Il-17a^-/-^* mouse splenocytes by flow cytometry. Under control conditions, there was an increase in the IFNγ^+^ CD4^+^ T cells in the *Il-17a^-/-^* mice as compared to the WT mice (Fig S6B). Accordingly, there was a significant increase in the proportion of LIGHT^+^ T cells in this subpopulation in the *Il-17a^-/-^* mice as compared to the WT mice (Fig S6B). Taken together, the data imply a relationship between LIGHT expression in Th1 CD4^+^ T cells, which is negatively regulated by IL-17A (lines:383-387)

Fig S6B

**RESPONSES TO COMMENTS BY REVIEWER NUMBER 2**

We thank the reviewer for his/her thoughtful and constructive examination of our manuscript. We have addressed all the reviewer’s concerns in detail. Our responses to the reviewer’s comments are as follows:

**COMMENT** 1. In some analyses, precise statistical evaluations are missing In Figure 1D–E, statistical comparisons are shown for WT vs WT-DDCfed and Il17A KO vs IL17A-DDCfed mice; however, it is unclear whether there is also a statistical difference between WT-fed and KO-fed mice. Additionally, in Figure 1B, the statistical significance is not clearly indicated.

**RESPONSE**. We did not observe any difference in the morphological or clinical parameters between the cholestatic WT and the *Il17a^-/-^* mice. This has been clarified in lines 235-236 of the manuscript. Gene expression of *Il17a* in intrahepatic leucocytes of mice was repeated and Figure 1b has been updated to indicate statistical significance.

**COMMENT** 2. Most of the figures are difficult to read due to small font sizes and low image resolution, making it challenging to interpret the data. There are general resolution problems in microscopic images. For instance, in Figure 1B, CK19 staining is barely visible, and the scale bars are inconsistent. A similar issue is observed in Figure 2C and several other figures. In some figures (e.g., Figure 5A–B, Figures S2–S3), the legends and labels are also hard to read.

**RESPONSE**. We thank the reviewer for pointing this out. All font sizes have been increased in the figures and panel legends. The brightness levels of the photomicrographs were enhanced to provide greater visual clarity. Additionally, in certain figures, some panels have been enlarged to provide greater resolution. These approaches have improved the quality of the figures.

Scale bars are different only in the tile scan images presented in Fig 2b but represent a uniform length of 1000 µm in each scanned image. This is because the tile scan images are stitched together from a varying number of smaller ‘tiles’ that covers the whole liver tissue section. As the tissue size differed from one slide to another, the software adjusted scale length to provide a comparable reference of 1000 µm for each composite image. The details of the method are described under materials and methods (lines 164-167). The legend has been updated to provide better clarity.

**COMMENT** 3. Previous studies have shown that LIGHT enhances TGF-β production and fibrotic gene expression via the LTβR–NF-κB pathway. However, this study does not examine whether LIGHT exerts its effect through this signaling route. Similarly, it remains unclear whether the STAT1 pathway was investigated, although it could be mechanistically relevant.

**RESPONSE**. We agree with the reviewer that previous studies have shown that LIGHT enhances *Tgf-β* production and contributes to fibrosis in multiple disease models. Mouse hepatic stellate cells express one of the receptors for LIGHT, LTβR (PMID 19111021) and given that NF-κB is known to regulate activation, survival and inflammatory response in fibroblasts (PMID: 21293511), it is likely that this signaling pathway is active in the DDC diet-induced liver injury model also. In the current study, we see significantly increased gene expression of *Tgfβ*, and multiple pro-fibrotic genes known to be regulated by NFκB (*Coll1a1, Pdgf, Acta2*) in the liver as well as in primary human fibroblasts. Thus, LIGHT does appear to induce NF-κB cognate gene targets suggesting activation of this pathway, and therefore we did not interrogate Stat1 involvement.

**COMMENT** 4. Furthermore, the observation that LIGHT elevation shifts polarization toward the T1 phenotype raises questions about which cytokines may mediate this process. For example, were IFN-γ or other related cytokines analyzed?

**RESPONSE.** We would like to clarify that we do not claim that LIGHT shifts polarization towards a Th1 phenotype, rather our observations are the reverse. Our data demonstrates that cholestatic liver injury induces the expansion of LIGHT^+^ in the CD4^+^ T cell subsets. For the latter comment on IFN-γ, please refer to our response to reviewe#1- comments 3 and 6.

**COMMENT 5**. The discussion section would benefit from a more integrative approach one that connects these possible mechanisms and situates the findings within the broader context of LIGHT-related studies. Specifically, the authors should discuss how T-cell polarization in the liver may be influenced by LIGHT, and how the NF-κB and STAT pathways could be involved in these interactions.

**RESPONSE.** We thank the reviewer for this comment. We have now carefully discussed our observations on LIGHT^+^ Th1 T cells in the context of DDC diet-induced cholestatic liver injury and the possible mechanisms regulating fibrosis.
