# Supplementary material for: Genetic ablation of interleukin-17A augments fibrosis in a mouse model of cholestatic liver injury
Source: PLoS One. 2026 Feb 6;21(2):e0342251. doi: 10.1371/journal.pone.0342251 (PMC12880643; doi:10.1371/journal.pone.0342251)
Supplement: Attachment — Submitted filename: PONE D 25.52210R1 RESPONSES TO COMMENTS BY REVIEWERs.docx [file pone.0342251.s012.docx]

**RESPONSES TO COMMENTS BY REVIEWER NUMBER 1**

We thank the reviewer for his/her thoughtful and constructive examination of our manuscript. We have addressed all the reviewer’s concerns in detail. Our responses to the reviewer’s comments are as follows:

**COMMENT 1.** Several additional points could further strengthen the manuscript, although are not critical for acceptance. **First**, the absence of in vivo loss-of-function experiments targeting LIGHT (e.g., antibody-mediated blockade) is understandable given practical limitations; while such experiments would provide stronger mechanistic support, their omission does not fundamentally compromise the main conclusions, which are presented in a suitably cautious manner.

**RESPONSE:** We thank the reviewer’s acknowledgement of the experimental limitations of the above-mentioned experiment and for appreciating that the results are presented with caution.

**COMMENT 2.** IL-17F is evaluated only at the transcript level. As mRNA expression does not necessarily reflect protein abundance and IL-17F could theoretically contribute to compensatory signaling, this limitation should be acknowledged more explicitly. Nevertheless, it does not materially alter the interpretation of the findings.

**RESPONSE:** We acknowledge that IL-17F was determined only at the transcript level. This has been addressed explicitly on lines 234-235 of the manuscript as follows: “The absence of compensation by *Il-17f* was also excluded by qRT-PCR for its mRNA, albeit this was not confirmed at the protein level (Fig. 1C).”

**COMMENT 3.** In addition, it would be important to clearly state as conclusion that the reported effects are specific to the DDC-induced cholestasis model and should not be generalized to other forms of liver injury or fibrosis without further validation.

**RESPONSE:** The conclusion of the abstract (lines 52-54) has been revised to state “Taken together, these data suggest that IL-17A restrains expression of the profibrogenic cytokine, LIGHT, by Th1-polarized CD4^+^ T cells, and implicate a role for LIGHT in cholestatic fibrogenesis in DDC-fed mice; a finding which requires validation in additional models.”

**COMMENT 4.** Finally, for clarity and transparency, it would be appropriate for the authors to explicitly state the number of biological replicates (n) in each figure legend.

**RESPONSE:** The number of biological replicates relevant to individual panels within each figure has been included in the legend.

**RESPONSES TO COMMENTS BY REVIEWER NUMBER 2**

We thank the reviewer for his/her thoughtful and constructive examination of our manuscript. We have addressed all the reviewer’s concerns in detail. Our responses to the reviewer’s comments are as follows:

**COMMENT 1**. The authors discuss their observations on LIGHT⁺ Th1 T cells in the context of DDC diet–induced cholestatic liver injury and fibrosis. However, the mechanistic aspects underlying these observations remain relatively limited. A more detailed discussion of the potential cellular and molecular mechanisms by which LIGHT⁺ Th1 T cells may contribute to fibrosis would substantially strengthen the Discussion.

**RESPONSE:** The discussion has been updated to include potential mechanistic aspects of the contribution of LIGHT to fibrosis. The discussion on lines 435-442 reads as follows: “This observation is consistent with observations reporting that the transcription factor NFAT promotes a Th1-like pattern of gene expression in primary murine CD4 T cells (PMID11994444), and that NFAT is known to enhance LIGHT expression (PMID 12215452). Moreover, LIGHT is known to induce TGFβ in macrophages which is pro-fibrogenic (PMID 37465675), and our studies demonstrate that LIGHT may directly activate primary HSC. Taken together, these data suggest that Th1-polarized CD4^+^ T cells may promote cholestatic fibrogenesis by expressing LIGHT. Whether LIGHT is sufficient to promote cholestatic fibrogenesis or whether it synergizes with other mediators as has been reported in pulmonary fibroblasts will require further delineation (PMID 40972652)”.

**COMMENT 2.** Gene names are inconsistently formatted throughout the manuscript (e.g., COL1a1 on page 10). Please ensure consistent and correct gene nomenclature according to standard conventions.

**RESPONSE:** We thank the reviewer for pointing this out. We have reviewed the manuscript and ensured that the gene nomenclature is per standard convention.

**COMMENT 3.** The resolution of some figures remains suboptimal. In particular, Figure 5b would benefit from higher resolution to improve readability. In Figure 3, the immunofluorescence signals appear weak and are difficult to distinguish. Improving image contrast and resolution, or providing higher-magnification images, would help readers better appreciate the findings.

**RESPONSE:** In Figure 5B, the figure size and font size have both been enlarged to improve the readability of individual gene names within the panel. In Figure 3, immunofluorescence in the red channel has been pseudo-colored to yellow to enhance contrast and improve visibility.
